# Smart control and management for a renewable energy based stand-alone hybrid system

**DOI:** 10.1038/s41598-024-83826-1

**Published:** 2024-12-30

**Authors:** Abdelhak KECHIDA, Djamal GOZIM, Belgacem TOUAL, Mosleh M. ALHARTHI, Takele Ferede AGAJIE, S. M.Sherif GHONEIM, Ramy N. R. GHALY

**Affiliations:** 1 Applied Automation and Industrial Diagnostics Laboratory, Ziane Achour University Djelfa, Djelfa, Algeria; 2 Renewable Energy Systems Applications Laboratory (LASER), Ziane Achour University Djelfa, Djelfa, Algeria; 3 Department of Electrical Engineering, College of Engineering, Taif University, Taif, 21944 Saudi Arabia; 4 Department of Electrical and Computer Engineering, Faculty of technology, DebreMarkos University, Debre Markos 269, Aksum, Ethiopia; 5 Chitkara Centre for Research and Development, Chitkara University, Baddi, 174103 Himachal Pradesh India; 6 Ministry of Higher Education, Mataria Technical College, Cairo, 11718 Egypt

**Keywords:** Renewable energy, Hybrid system, MPPT, ANFIS controller, Management, Engineering, Electrical and electronic engineering

## Abstract

This paper addresses the smart management and control of an independent hybrid system based on renewable energies. The suggested system comprises a photovoltaic system (PVS), a wind energy conversion system (WECS), a battery storage system (BSS), and electronic power devices that are controlled to enhance the efficiency of the generated energy. Regarding the load side, the system comprises AC loads, DC loads, and a water pump. An Adaptive Neuro-Fuzzy Inference System (ANFIS)-based MPPT technique is suggested to enhance the efficiency of the PVS and WECS. This technology provided good performance compared with the Perturb and Observe (P&O) algorithm and MPPT-based fuzzy logic controller (FLC). The use of the ANFIS-PI proposed to control the bidirectional converter accomplished voltage stabilization for the DC bus. This work also came with a fuzzy logic-based algorithm to manage the load side that depends on battery charge ratio, solar radiation, and wind speed. According to results obtained in the MATLAB/Simulink environment, the proposed technologies were found to have performed well. The goal we were also pursuing was achieved through the full use of the energy generated by the proposed algorithm. The proposed study holds great potential for remote regions.

## Introduction

Recently, the adoption and use of renewable energy sources has witnessed remarkable growth. This is due to a decrease in the global reserve for fossil fuels^1^. The latter significantly impacts ozone layer expansion and environmental pollution. The economics of fossil resources have also become expensive due to the high energy demand. Renewable resources can be exploited in a small or stand-alone networks to use these systems for agricultural applications and support isolated areas such as military barracks, islands, and even rural areas away from electrical grids^2^. The high cost and environmental impact of polluting energy sources have led to renewable energy becoming essential for electricity production^3,4^.

### Motivations

Thanks to their availability, simplicity of use, and clean environmental properties, solar and wind energy are among the most reliable sources of renewable energy^5,6^. Due to the intermittent and unpredictable nature of photovoltaic and wind generators and the variable load demand, energy storage system integration in systems based on renewable sources is essential^7^. By strategically placing an energy storage system, can enhance the quality of energy by regulating frequency and voltage. This will also reduce the impact of fluctuations and increase the value of the existing supply, especially during high electricity usage periods^8,9^. Renewable energy systems that are autonomous can be used on either an AC bus, a DC bus, or a combination of both. It is simpler to manage a DC bus because of its uncomplicated structure and lower information requirements. However, an AC bus must be closely designed and demands high information accuracy, like frequency synchronization and interactive power. This can make it hard to control, but an AC bus may be necessary in certain situations^10^.

Literature Review.

With the latest developments in Electronics related to energy. the independent hybrid system can now function at its maximum potential. Nevertheless, since renewable resources can be erratic, a supplementary power management unit must ensure seamless operation and uninterrupted power supply to loads. Several research studies are accessible on energy management control for autonomous access, which can be located in literary sources. Numerous techniques have been devised to enhance the efficacy of renewable energy systems, alongside developing strategies for effectively managing hybrid renewable energy systems. In^11^the energy management system was implemented for a stand-alone hybrid system with two sustainable energy sources: wind, solar, and battery storage. To monitor maximum energy points efficiently, the P&O algorithm was used to control photovoltaic and wind power systems. The battery storage system is organized via PI controller. This study aimed to improve the energy quality and ensure that the optimal voltage level is maintained. In^12^, A strategy for energy management of water pumping systems is designed for agricultural applications. This system adopts solar power and a storage battery to power the DC motor. Where the P&O algorithm MPPT was modified to improve the system’s effectiveness, this algorithm was proposed to solve the problem of drift of photovoltaic accretion due to the random nature of solar radiation. In^13^, The authors presented an energy management strategy for an independent system, drawing on a fuzzy logic approach. The work is centred on the use of wind and photovoltaic power. A hybrid storage system combines a Battery Storage System and a Super Condenser (SC), Diesel generator (DG) has been used as an emergency power depletion measure and insufficient generating sources.in^14^, Researchers have rolled out an independent hybrid system consisting of PVS, WECS, diesel generators (DG), and BSS. This study introduces a new approach to improve power generation in photovoltaic systems, as the FUZZY-PI controller is integrated with the P&O algorithm. In addition, the supervisory control algorithm is designed to regulate power flows within the proposed methodology effectively. In^15^, The application of artificial neural network (ANN) technique has been employed in solar and wind systems, respectively, to enhance the overall efficiency of these systems. The objective of this study is to enhance the efficiency of battery utilization and optimize management in a hybrid power system. In^16^, A pilot study of a stand-alone hybrid system consisting of PVS, fuel cells (FC), wind power conversion systems, and (BSS) was presented. Individual speed and logic controllers have been used to improve the MPPT. The energy management technique used in this study was based on assessing battery charging status. As for maintaining the current symmetric voltage, Fractional-order-PID has been relied upon. In^17^, Researchers put forward a proposal for a micro-DC network based on renewable energy sources. To support the electric vehicle charging system. This study focuses on developing a particle swarm improvement algorithm (PSO) to manage the system, balance sources and loads, and rapidly regulate the carrier’s ongoing efforts. In contrast, concerning hybrid energy systems (HES) that are interconnected with electrical grids, in their publication, referred to as^18^, the authors introduced a hybrid system integrated with the electrical grid. This study focuses on developing and analysing three distinct energy management methods, namely FLC, ANN, and PI. The control and organization of the bidirectional converter DC/DC have been facilitated through the use of several techniques. The results of this study showed disparities between the proposed techniques. In a previous study of^19^, different methodologies were developed for managing a hybrid network system. This work involves using a sliding mode controller, a synthetic neural network, a proportional integrative derivative, and fuzzy logic. The researchers conducted experiments to assess the effectiveness of all working controllers and explained the advantages and limitations of each methodology. However, it is essential to conduct a pilot study to verify the results. In^20^, the study presented a photovoltaic system associated with the electricity grid. A combination of storage battery and diesel generator supports the plan. voltage source converter (VSC) was used to connect photovoltaic (PV) sources, storage systems, with AC network. The balance between load energy and power generation in island mode was achieved using a diesel generator. The storage system played a critical role in mitigating volatility and helping the system mitigate errors. A new model for AC-DC hybrid micro-networks utilizing renewable energies was introduced in a previous study^21^. According to their research, renewable energy sources have a significant role in cost reduction and load support during service. For the optimal effectiveness of photovoltaic systems, the maximum control unit is used for the purpose of monitoring the maximum power point. Several techniques MPPT for photovoltaic systems have been proposed in the literature. The algorithm P&O was used in^22–24^. However, due to technical advances, this method has become less used due to its inherent flaws in oscillations at the MPPT and deviations at the whole operating point, especially in changing weather conditions. To alleviate the constraints of these approaches, scientists have created artificial intelligence (AI) technologies based on MPPT to address this problem. In^25^, The authors proposed a charging station for electric cars powered by solar energy and supported by storage batteries. In this work, an improved cuckoo search algorithm (CUSA) was relied upon to track the maximum power point. The effectiveness of the proposed technique was verified in five different scenarios. The researchers in reference^26^utilized the MPPT technique with FLC to investigate the performance of a photovoltaic system across varying solar radiation levels and temperature conditions. In^27^, The authors presented the design of a 200-watt PVS, where the FL-DPID MPPT technology was implemented and compared with other technologies. The performance of this technology was tested in four different cases of solar radiation, and the results of this paper showed good performance in terms of voltage and current output as well. This technology is characterized by effective and fast tracking. The study of^28^presents a proposed approach for the design of fuzzy logic controller transactions, utilizing the PSO algorithm, to maximize power tracking in photovoltaic systems. The authors^29^compared the fuzzy logic algorithm and the disorder algorithm and surveillance. The study results indicate that the FLC-based MPPT system shows a faster convergence with the maximum power point compared to the P&O algorithm. Nevertheless, a significant drawback of the FLC unit is the issue of drift, which arises due to the influence of solar radiation and temperature. This problem is effectively addressed by the utilization of ANFIS technology by researchers. The ANFIS-based MPPT technology exhibits superior response time and reduced oscillation compared to the P&O and FLC methods. In^30^, ANFIS-based MPPT technology was trained using actual data from the electrical system. Researchers conducted a comparative analysis of ANFIS technology, fuzzy logic technology, and traditional technologies, and the results of their study show high efficiency and high performance compared to other technologies. The authors^31^presented a study on the design and analysis of solar system applications using MPPT-based ANFIS. The training of this unit was conducted using data collected during the year 2018, with the purpose of minimizing the system’s susceptibility to significant errors. The aforementioned experience has demonstrated remarkable efficiency, surpassing 99.3%. In^32^ the authors provided a comparison between the use of FLC and ANFIS technology, where the results of this work proved that ANFIS outperformed FLC in tracking speed.

1.3. Research Contributions.

Based on existing scholarly literature, it is commonly observed that many writers employ either grid connections or diesel generators to maintain a consistent energy supply inside systems reliant on renewable energy sources. However, perfect energy management can make the self-based green hybrid system ensure a constant flow of energy in various conditions. This study aims to enhance and improve the performance of the hybrid system. And smartly manage this system. The main contributions to this paper are set out below.

1 The implementation of green hybrid systems has the potential to make a significant contribution towards environmental preservation by mitigating the release of damaging gas emissions into the atmosphere.

2 New MPPT technique has been proposed and developed to improve the effectiveness and performance of the PVS and WECS.

3 Improved storage system performance. And improve its efficiency and control in a smart way using ANFIS-PI.

4 Intelligent energy flow management for various possible scenarios.

5 Propose an algorithm for load-side management (AC load and DC load and water pumping) and make full use of the energy produced from renewable sources.

The following parts of the paper were drafted as follows: Section II provides a comprehensive overview of the proposed system. The proposed controls and techniques are displayed on the power electronics interfaces in Section III. Section IV presents the proposed strategy for energy flow management. Section V presents the simulated results. The conclusion of this work is contained in Section VI.

## Description of the Studied System

The suggested design for a standalone hybrid power system involves incorporating two systems: PVS and WECS. A storage system serves as support, along with multiple electronic power devices such as converters, inverters, and bidirectional converters. This combination offers both technical and economic benefits in supplying energy to isolated regions or far from the electrical grid.

**Fig. 1:**
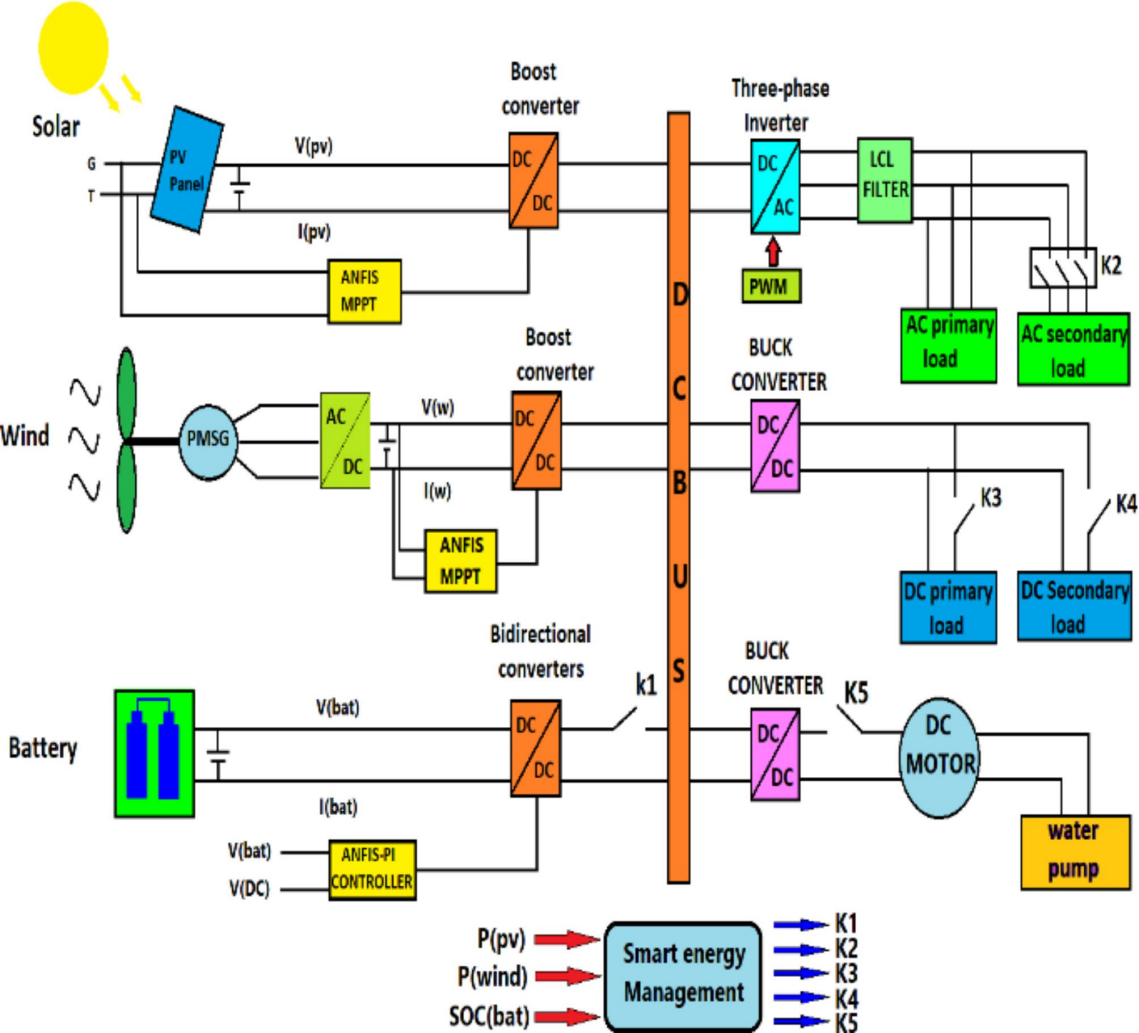
Structure of the stand-alone hybrid renewable energy system.

PVS includes a set of PV panels, and DC /DC converter, and a new intelligent MPPT controller. It is performed to get the maximum power generated from the photovoltaic system by tuning the boost converter working cycle. The WECS system utilizes a wind turbine and permanent magnetic synchronous generator (PMSG) for its configuration. It is controlled through a rectifier bridge for AC-DC and DC-DC converter, with an enhancement converter controlled by an intelligent MPPT to optimize the amount of power generated by the wind turbine.

The ESS system is designed to store excess energy generated by renewable sources and use it to cover any deficits in power supply. It comprises a battery bank and a DC-DC bidirectional converter connected to the DC bus. ANFIS technology intelligently controls the voltage to remain constant at the DC bus. The inverter then converts the DC power into AC power, which is used to power AC loads. The AC power is filtered through the LCL filter to ensure good power quality. Figure 1 shown the Structure of hybrid system proposed. and Tables 1, 2 and 3, and 4 present the component characteristics of the system studied.

**Table 1:** PVS specifications.

Parameter	Value
Maximum Power W	250.92
Open circuit voltage Voc (V)	37.4
Short-circuit current Isc (A)	8.7
Voltage at maximum power point Vmp(V)	30.6
Current at maximum power point Imp (A)	8.2
Parallel strings	4
Series per string	10

**Table 2:** PMSG Specification.

Parameter	Value
Stator phase resistance Rs (Ohm)	0.5
Armature inductance L (H)	0.000975
Torque constant Kt	60.7
pole pairs P	4
viscous damping F ( oz.in/krm)	4.5
Inertia J (kg.m²)	50

**Table 3:** Battery specifications.

Parameter	Value
Nominal voltage (V)	220
Rated capacity (Ah)	40
Internal resistance R( Ω)	0.04
Nominal discharge current (A)	21.73
Fully charged voltage (V)	232.8

**Table 4:** Converters specifications.

Parameter	Boost converter	Bidirectional converter
capacitor	1.850 m F	1.27 m F
inductor	1.123 m H	0.61 m H
switching frequency	20 K Hz	20 K Hz

##  Smart control of power electronics interfaces

Random changes in solar radiation, wind speed, and variable loads can distort the quality of energy produced from renewable sources. Therefore, MPPT is relied upon to overcome this obstacle. In this work, we developed an MPPT technique-based ANFIS for optimal control of DC/DC converters for PVS and WECS. Due to the unpredictable nature of these sources, the use of a storage system is necessary. Therefore, an ANFIS controller was proposed to control the DC/DC bidirectional converter intelligently.

### General overview of ANFIS

**Fig. 2:**
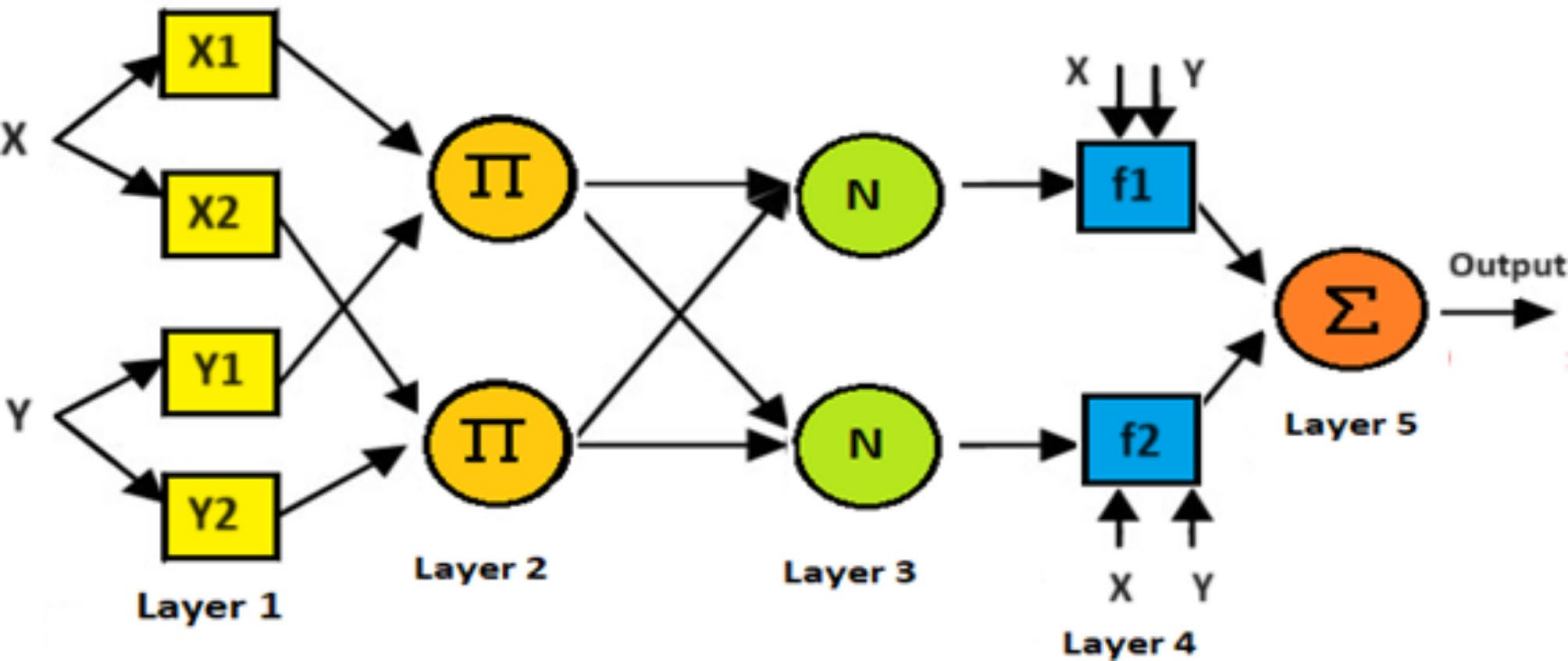
Structure of ANFIS.

ANFIS is a hybrid system that combines ANN and FLC in order to take advantage of the advantages of both technologies. ANFIS is used in various applications, including MPPT for photovoltaic system and wind system, and is also used as a voltage regulator and current in bidirectional converters of the storage system. Rules, Fuzzification, normalization consequent and addition are the five layers that make up the ANFIS architecture as shown in Fig. 2. Every node in the training dataset is an adaptive node, employing the node function as defined by Eqs. (1) and (2):

1 $$A1,i=uxi\left( x \right)$$

for i = 1,2 

2 $$A1,i=uy(i - 2)\left( y \right)$$

for i = 3,4 

where U denotes the specified membership function and A1,i represents the designated membership value for the inputs x and y.

Based on a single fuzzy rule, each node in Layer 2 is fixed. Equation (3) gives the output value:

3 $$A2,i=\omega i=uXi(x)uYi\left( y \right)$$

for i = 1,2 

Using Eq. (4) to normalize the firing strength, each node in Layer 3 is fixed.

4 $$A3,1=\frac{{\omega i}}{{\omega 1+\omega 2}}$$

Each node in Layer 4 is modified and calculated according to the rule consequent, as specified by Eq. (5):

5 $$A4,1=\omega ifi=\omega i(pix+qiy+ri)$$

pi, qi, and ri are sequential parameters that necessitate optimization during the training process.

The ultimate output signal in Layer 5 is obtained by adding all of the input nodes together, as shown in Eq. (6):

6 $$A5,1=\frac{{\sum\nolimits_{i} {\omega ifi} }}{{\sum\limits_{i} {\omega i} }}$$

**Fig. 3:**
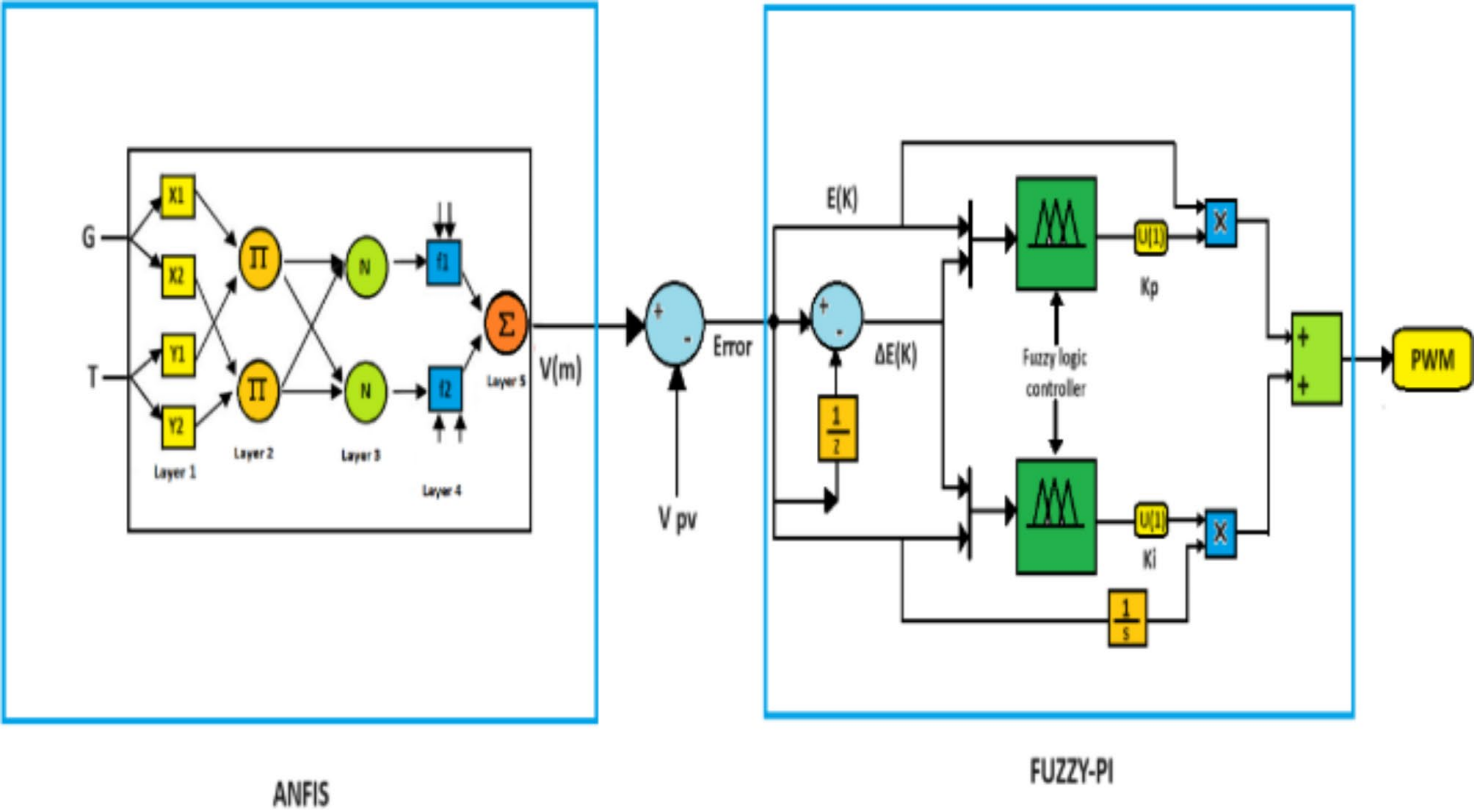
Structure of PV-MPPT proposed.

### PVS MPPT proposed

**Fig. 4:**
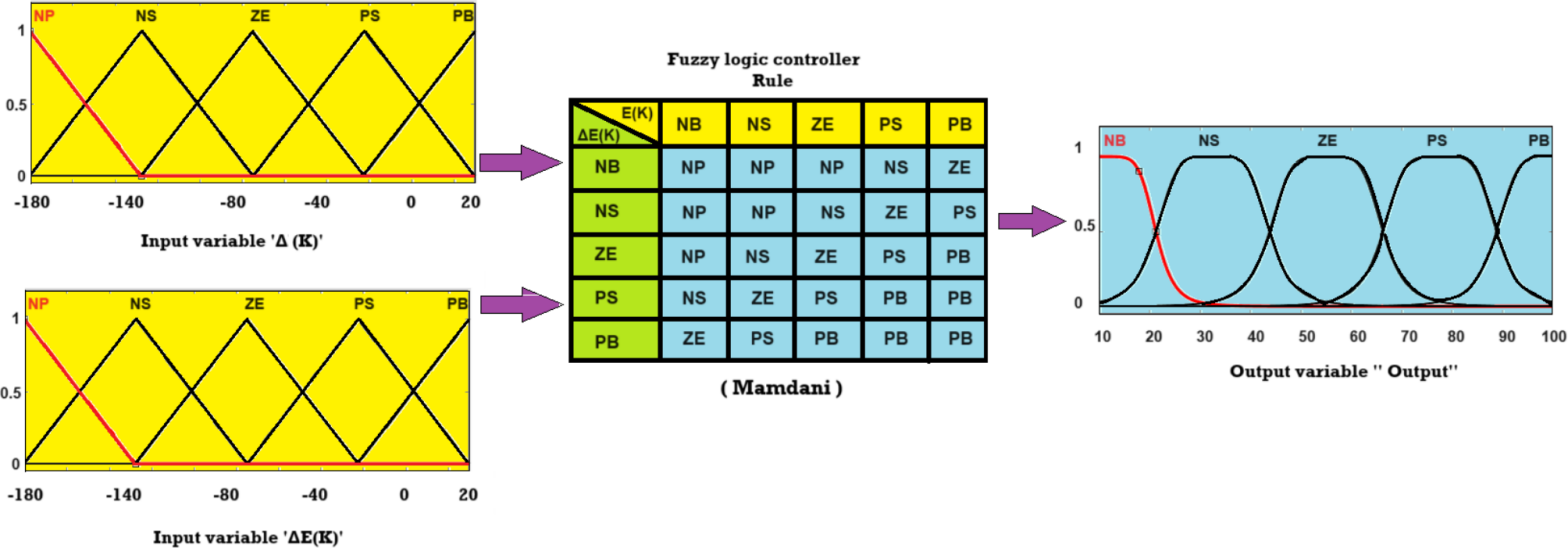
Fuzzy logic controller parameter for PVS.

**Fig. 5:**
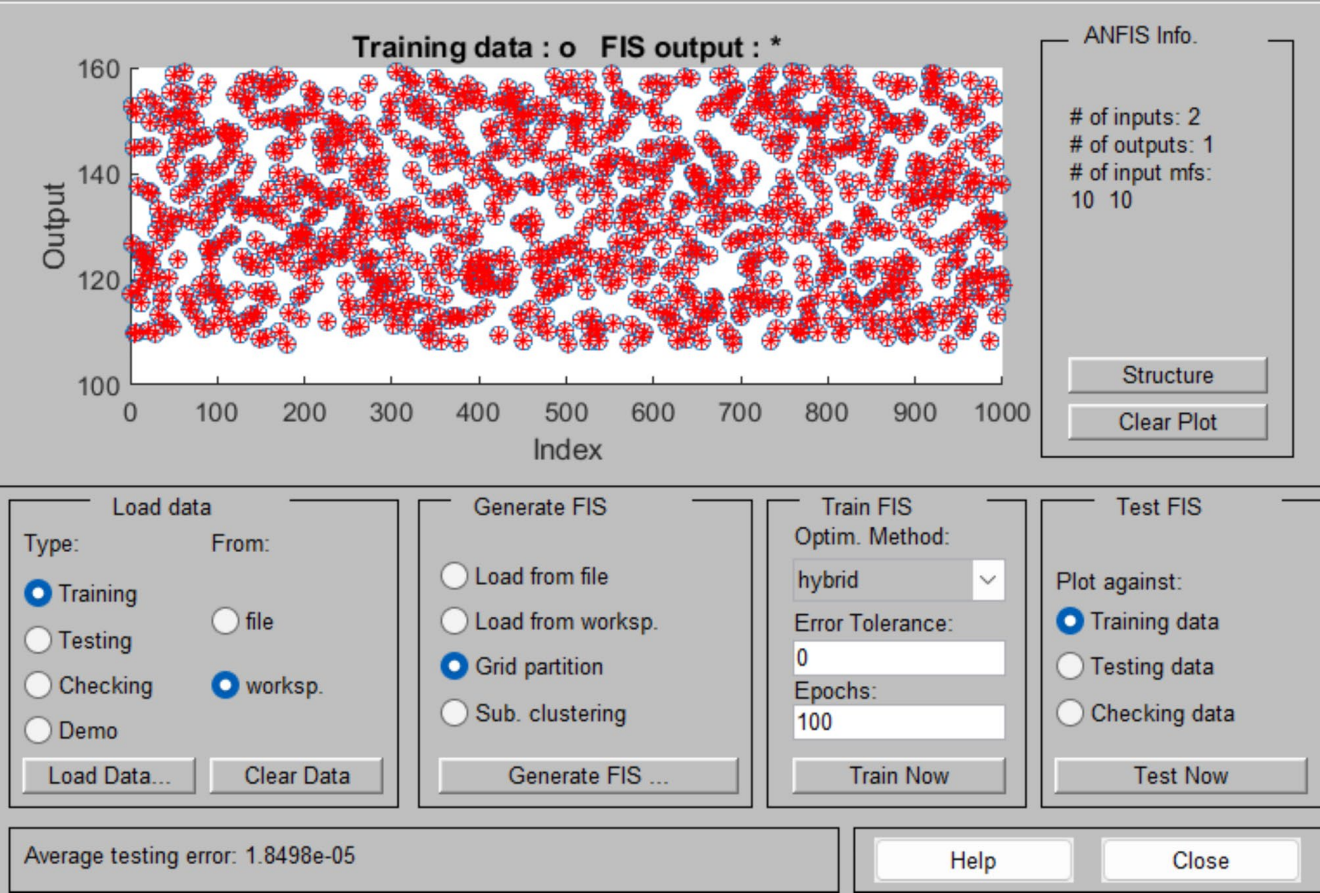
ANFIS controller parameter for PVS.

There are two components to the suggested MPPT framework., as Fig. 3 shows, the first part is based on the ANFIS technique. This technique receives solar radiation and temperature as input data, while the output is defined as the most comprehensive VM voltage that is created. This voltage is compared with the voltage of the photovoltaic array (Vpv), and then the result of this comparison is sent to the second part of the MPPT structure, which consists of the fuzzy-pi controller. Error and change of error are considered inputs to fuzzy-pi, and the duty cycle is its output. Figures 4 and 5 shows the parameters of FL and ANFIS controllers.

### WECS-MPPT proposed

**Fig. 6:**
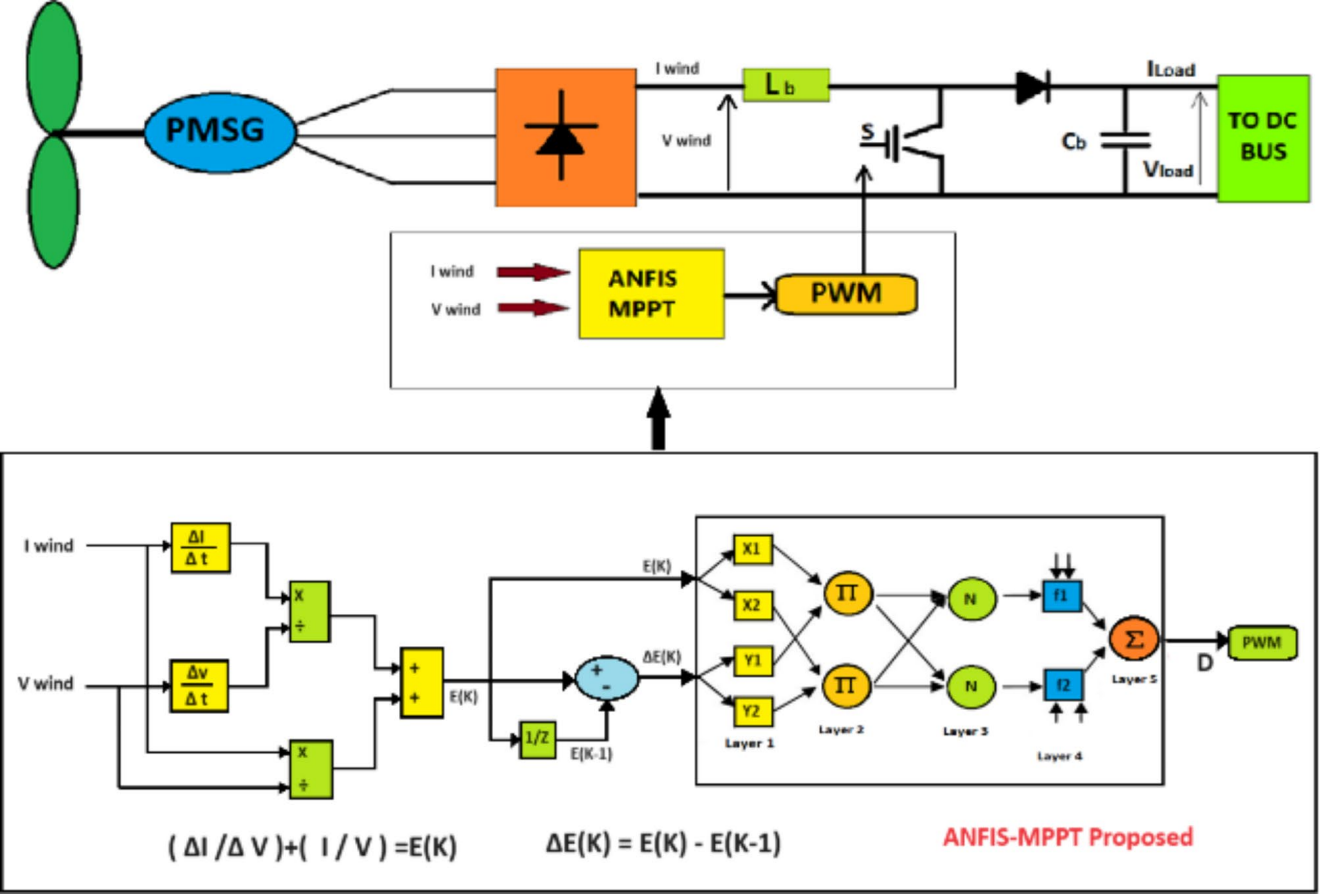
Structure of WECS control.

**Fig. 7:**
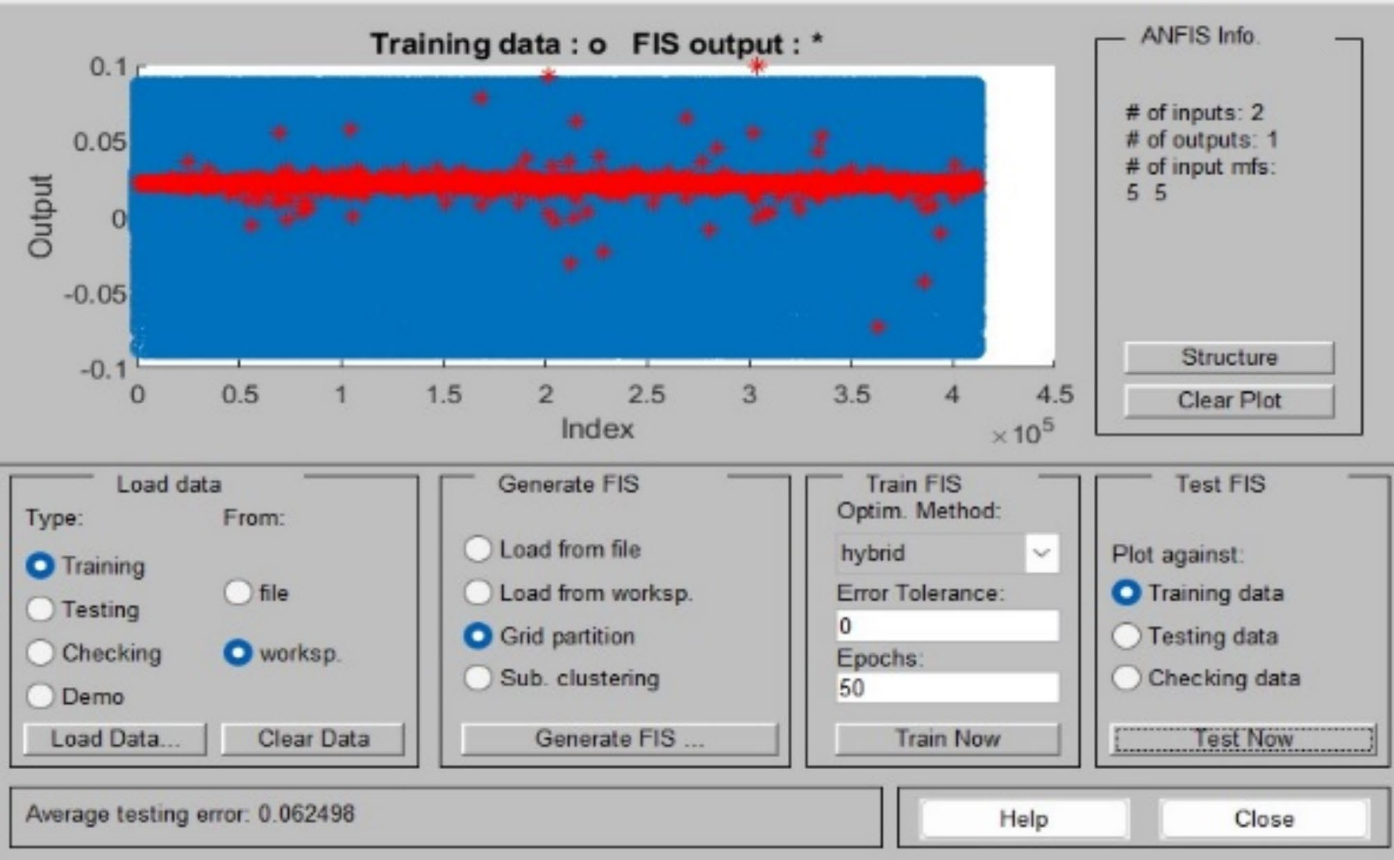
ANFIS controller parameter for WECS.

In order to optimize the energy output of a wind system, we suggest utilizing an MPPT method grounded in ANFIS. the error and change in error were considered inputs to the ANFIS, while the output was defined as the duty cycle. Numerous datasets were collected under various wind speed conditions, including the voltage and current produced by the PMSG. These datasets were then used to train the ANFIS, Fig. 6 shown the MPPT structure and Fig. 7 shown parameters of ANFIS controller.

### Storage system control

**Fig. 8:**
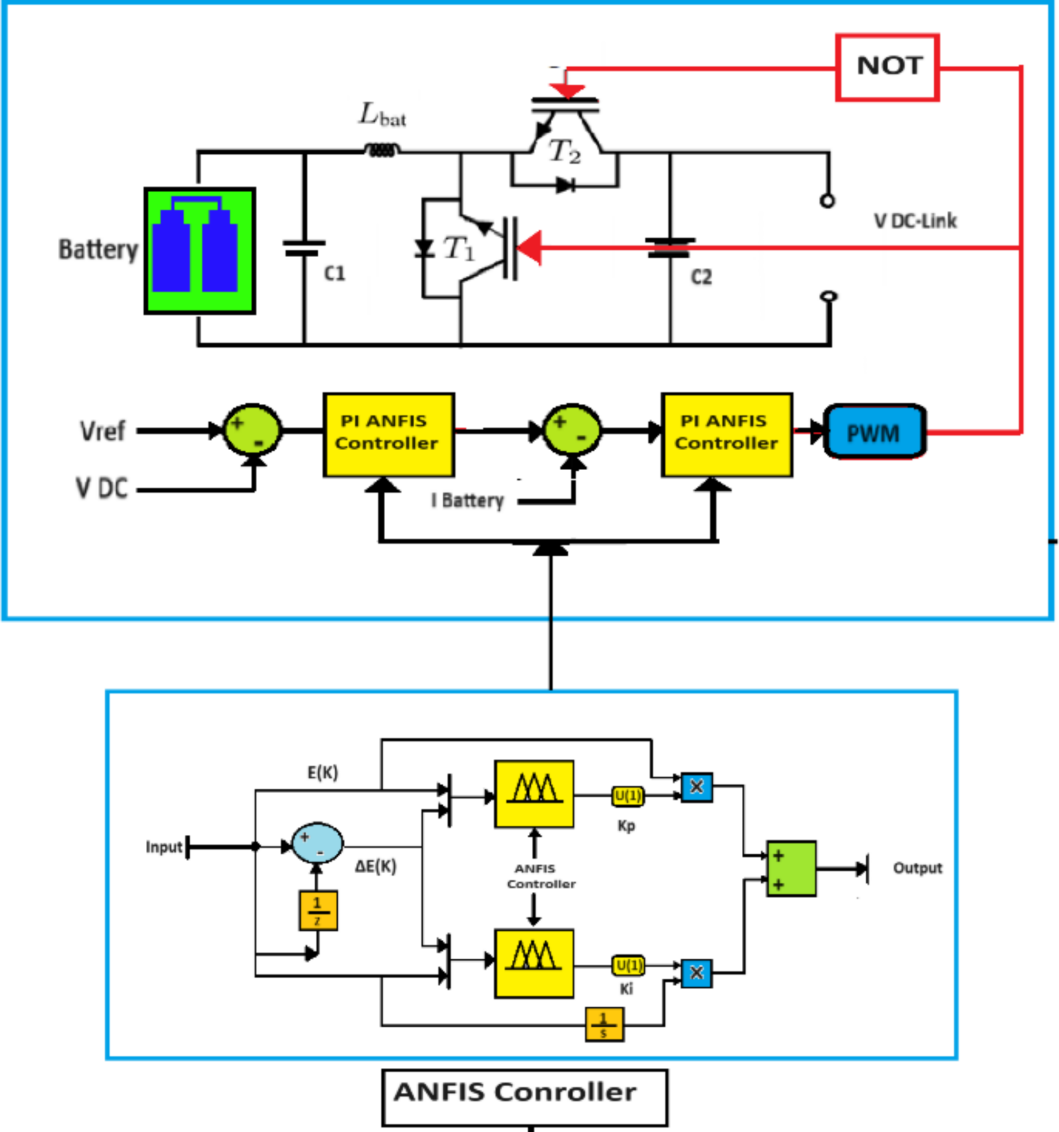
Proposed Bidirectional Converter controller.

Due to the random nature of renewable energy sources, the continuous flow of energy all the time is impossible. Therefore, integrating a storage system is necessary in order to ensure the continuous flow of energy to the loads. A bidirectional DC/DC converter is usually used for control and management the power flow in the system. This converter is controlled by generating a PWM signal. This signal is obtained by comparing the reference voltage and the DC bus voltage in order to obtain a reference current to compare the latter with the battery current and obtain the PMW. A PI controller is often used to generate PWM, but as is known, the PI is considered inappropriate, in nonlinear systems, because it is characterized by it has obvious disadvantages such as overshoot ratio and slow response. In order to fill these gaps, we proposed an intelligent controller based on ANFIS. Figure 8 shows the model of the proposed controller. We can see from the figure that the error and the change in error were considered as inputs, while the parameters Ki and Kp were considered as outputs. Where we trained the PI controller in different operating conditions and trained it on ANFIS. The ANFIS continuously adjusts the Ki and Kp values to improve the system’s response in different conditions. Thus, we have an intelligent system that can automatically improve itself and continuously adjust its parameters to achieve optimal performance.

## Energy management system

The supervisory control unit’s primary function is to ensure that the demand for loads is consistently met in the face of different weather conditions. The current study used an obscure logical controller to regulate energy distribution within the proposed system. The system consists of electricity-producing sources comprised of wind turbines, solar panels, and storage batteries. These loads are divided into essential loads and secondary loads. The proposed control unit has double access points. The initial entry relates to the cumulative power of renewables (wind and solar). Subsequent entries refer to the battery state of charge (SOC). Output: The unit’s total output is five. It is a K1 battery switch, a K2 AC secondary load switch, a K3 DC base load switch, a K4 DC secondary load switch, and a K5 water pump switch. The first entry contains seven organic functions. The second entry includes four organic parts. For outputs, they all have two organic posts (OF ON). The previously mentioned details can be succinctly summed up as follows:

• Scenario 1: (20kw > P gen > 18kw), all loads will be engaged. In the context of water pumping, prioritizing water pumping is typically regarded as a lower priority. The triggering event occurs when the battery charge ratio exceeds 85%. Once the battery has reached a charge level of 85%, it undergoes detachment from the system.
• Scenario 2: (18kw > P gen > 16kw), All loads are operated except for water pumping. If the battery charge ratio is less than 85%, the DC2 is turned off until the battery is charged. If charged more than 85%, it is separated from the system.
• Scenario 3: (16kw > P gen > 14kw), DC1, AC2, and AC1 are operated. The DC2 and the water pump were stopped. If the battery charge ratio exceeds 50% the previous loads keep operating. If the battery charge ratio is lower than that (50%), AC2 is stopped. And battery start charging up until it exceeds 50%.
• Scenario 4: (14kw > P gen > 12kw), The DC1 AC1 is activated. If the battery charge ratio is greater than 50%, the DC2 will be activated. If the charge ratio is less than 50, the DC2 is separated, and the battery charge continues.
• Scenario 5: (12kw > P gen > 10kw), AC1 and DC1 are operated. The battery is discharged when needed until the charge ratio reaches 40%, after which the battery is separated.
• Scenario 6: (10kw > P gen > 8kw), The AC1 DC1 is operated, and the battery discharges up to 40%. The DC1 is then separated. Then the battery is charged until the charging ratio reaches 50%.
• Scenario 7: (8kw > P gen > 0kw), Only AC1 operated. And discharge from the battery until the 20% ratio is reached, then the battery is separated.
• Scenario 8: (P gen = 0Kw), Open AC1 only. Discharge the battery up to 20%. And then the system stopped.

## Simulation results and discussion

*A. Improving the Performance of the hybrid system*.

**Fig. 9:**
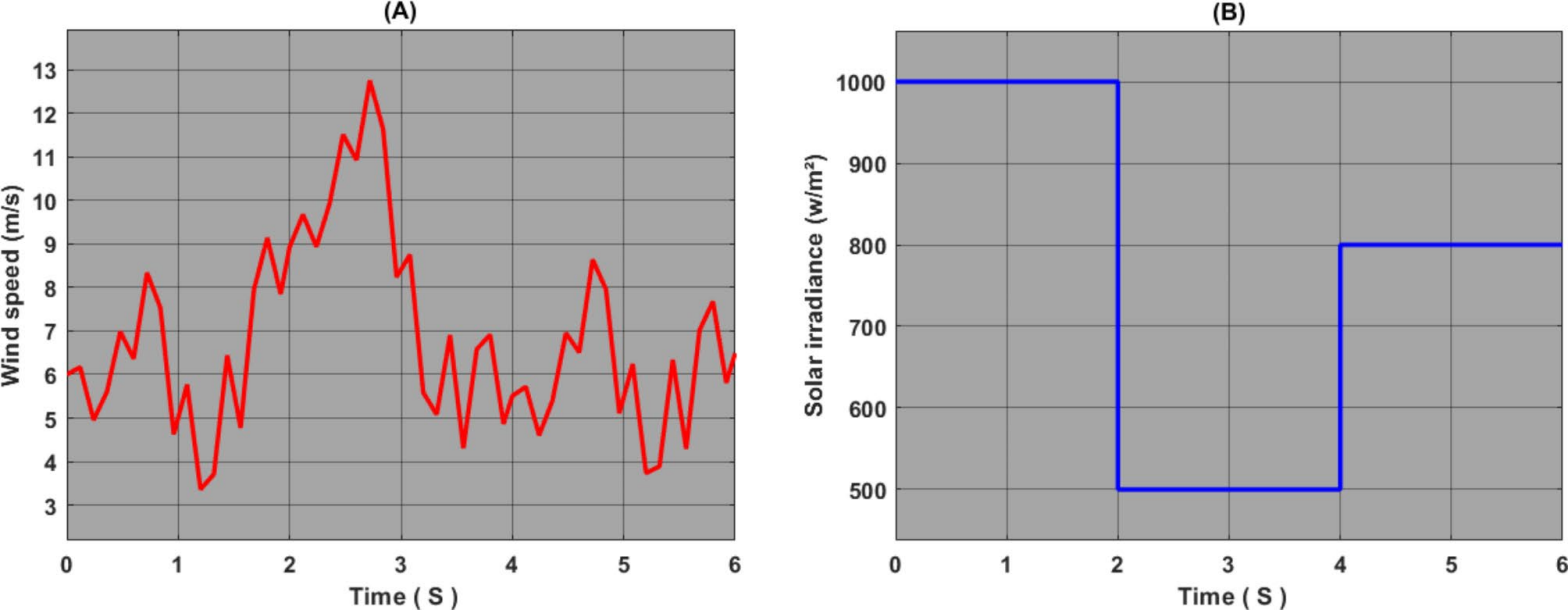
Proposed meteorological parameters: (a) wind speed, (B) sun radiation.

In this work, we simulated a standalone hybrid power system based on renewable energy sources using MATLAB/Simulink software. To enhance the PVS and WECS systems’ performance, we have developed an AI-based MPPT controller to raise the effectiveness of the energy produced from the two previously mentioned sources. In order to verify the effectiveness and performance of the proposed technique, we compared it with other techniques, namely: P&O, FLC, PSO and ANFIS; for the photovoltaic system, and the wind system it was compared with P&O, FLC and PSO. To reach a more accurate study, the simulation process was performed under different conditions of solar radiation and wind speed. Figure 9 shows the solar radiation and wind speed used in this work.

The loading profiles for solar and wind energy were chosen based on the following criteria:

Real wind speed to test the efficiency of the wind system in all weather conditions and compare the various techniques studied^33^.

Different solar radiation values ranging from 1000 w/m² to 500 w/m² to verify the efficiency and performance of the proposed approach.

**Fig. 10:**
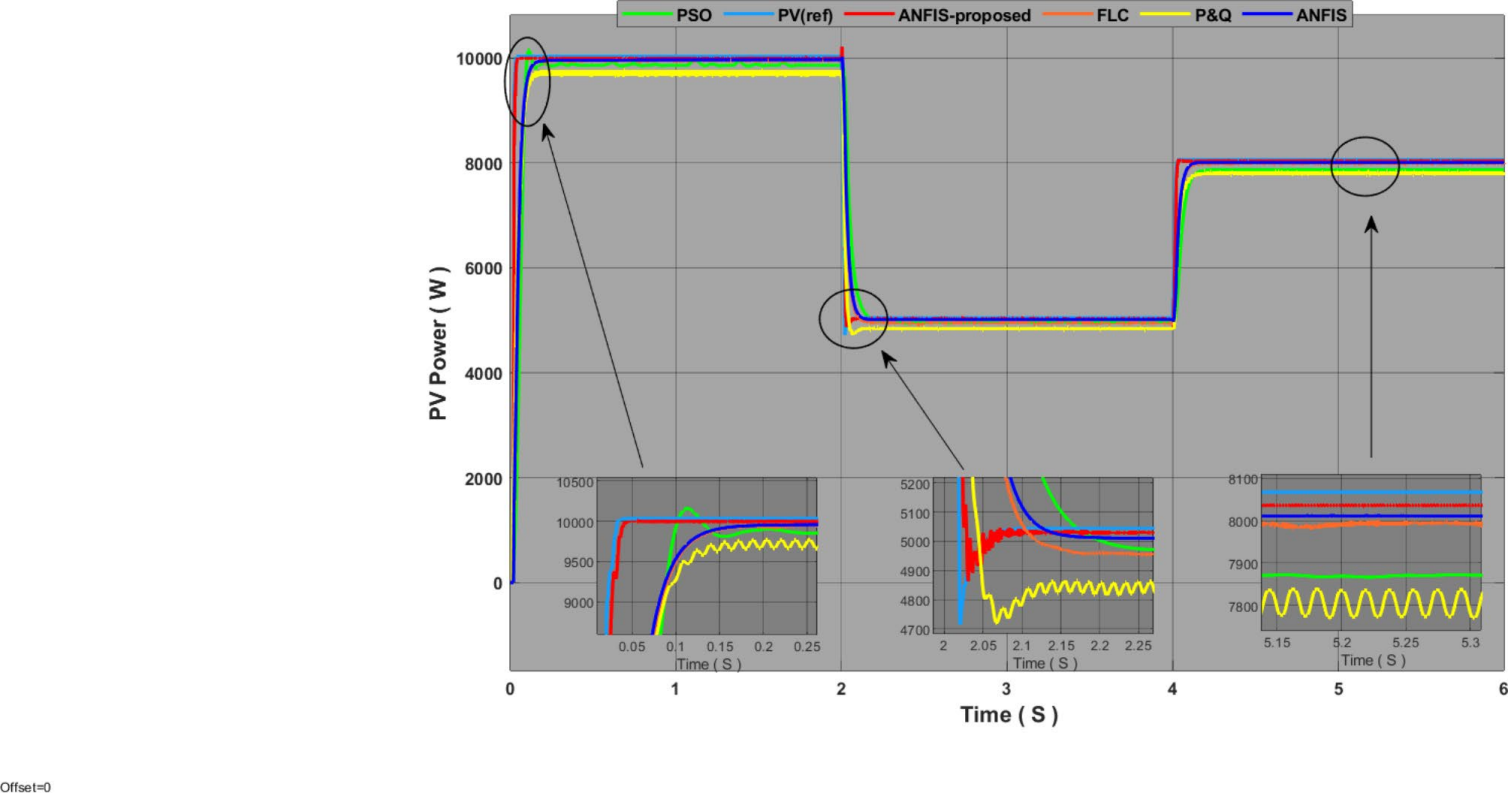
Performance comparison of the proposed MPPT with other technologies for PVS.

Figure 10 shows the PVS simulation results in variable solar radiation conditions and constant temperature at 25°. The results were analysed by critically comparing the proposed technique with other methods according to system response time, oscillation capacity, and optimal energy production. We can say that P&O’s algorithm is the weakest technique in terms of performance and effectiveness, as results show us that the response time for this technique is slow with significant fluctuations and the maximum point of energy was the lowest among all technologies, all of which translate into substantial losses at energy outputs. In contrast with P&O, the PSO technique offers a quicker response time, fewer oscillations, and more production effectiveness. The FLC method has a fast response time and good production effectiveness but a medium oscillation capacity, which is what doesn’t make it optimal technique. ANFIS technology performed better than other technologies in terms of production effectiveness and oscillation ratio, but the response time was less than needed. With regard to the proposed technique ، the results showed that the proposed approach was higher in effectiveness and performance than the other techniques mentioned, with a very rapid response time of 0.05 (S), an oscillation ratio that was almost non-existent, and an excellent production effectiveness of 99.75%. Further comparisons and analyses are shown in Table 5.

From the results of this experiment, we can say that the proposed MPPT is more efficient, and has a better performance, which allows for the improvement of the productivity of the photovoltaic system.

**Fig. 11:**
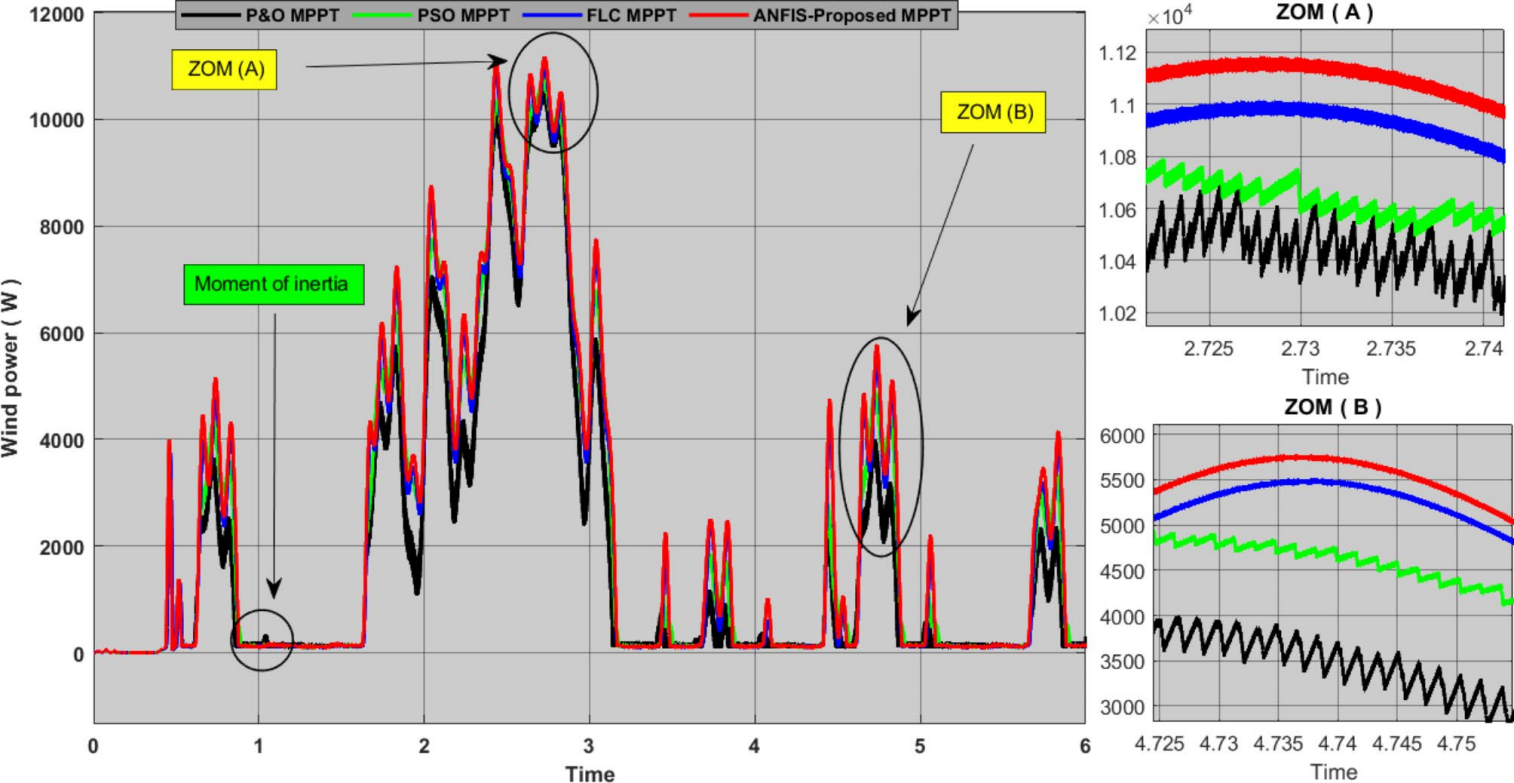
Performance comparison of the proposed MPPT with other technologies for WECS.

Figure 11 shows the simulation results of a wind system representing the output of energy produced by different technologies with actual wind speeds ranging from 3.5 to 12.5. According to the results obtained from the different technologies applied to the system, the traditional P&O algorithm imposes a significant energy loss due to its low efficiency and slow response time with a large oscillation ratio, especially at weak wind speeds, this makes performance very poor. While the PSO technique has a medium oscillation rate with a better production efficiency compared to the P&O algorithm, with a medium response time. The FLC method has good production efficiency and almost no oscillation rate, but the weakness of this technique is the response time, where we see it as the slowest of all techniques. As for the proposed approach, it turned out to be optimal out of all the techniques applied to the system. We can note that it has a fast response time, no oscillation, and excellent production effectiveness. Through these dynamics, we can say that the proposed approach increases production efficiency and improves the wind system’s energy quality. Table 5، shows more detailed analyses and comparisons of this simulation results.

**Table 5:** Contrasting the suggested MPPT’s performance and efficacy with that of other MPPTs.

MPPT	oscillation		Response time		Effectiveness	
	PV	WIND	PV	WIND	PV	WIND
P&O [23]	High	High	0.15 (S)	0.1 (S)	96%	92%
PSO [28]	Medium	Low	0.10(S)	0.09 (S)	98.2%	95%
FLC [29]	Low	very low	0.15(S)	0.15 (S)	99%	98%
CUSA [25]	Low	--------	0.05	------	99.26	------
FL-DPID [27]	Low		0.02	-------	99.65	------
ANFIS [31]	very low	---------	0.11(S)	-----	99.3%	-----
Our MPPT	very low	very low	0.04(S)	0.05 (S)	99.7%	99.1%

**Fig. 12:**
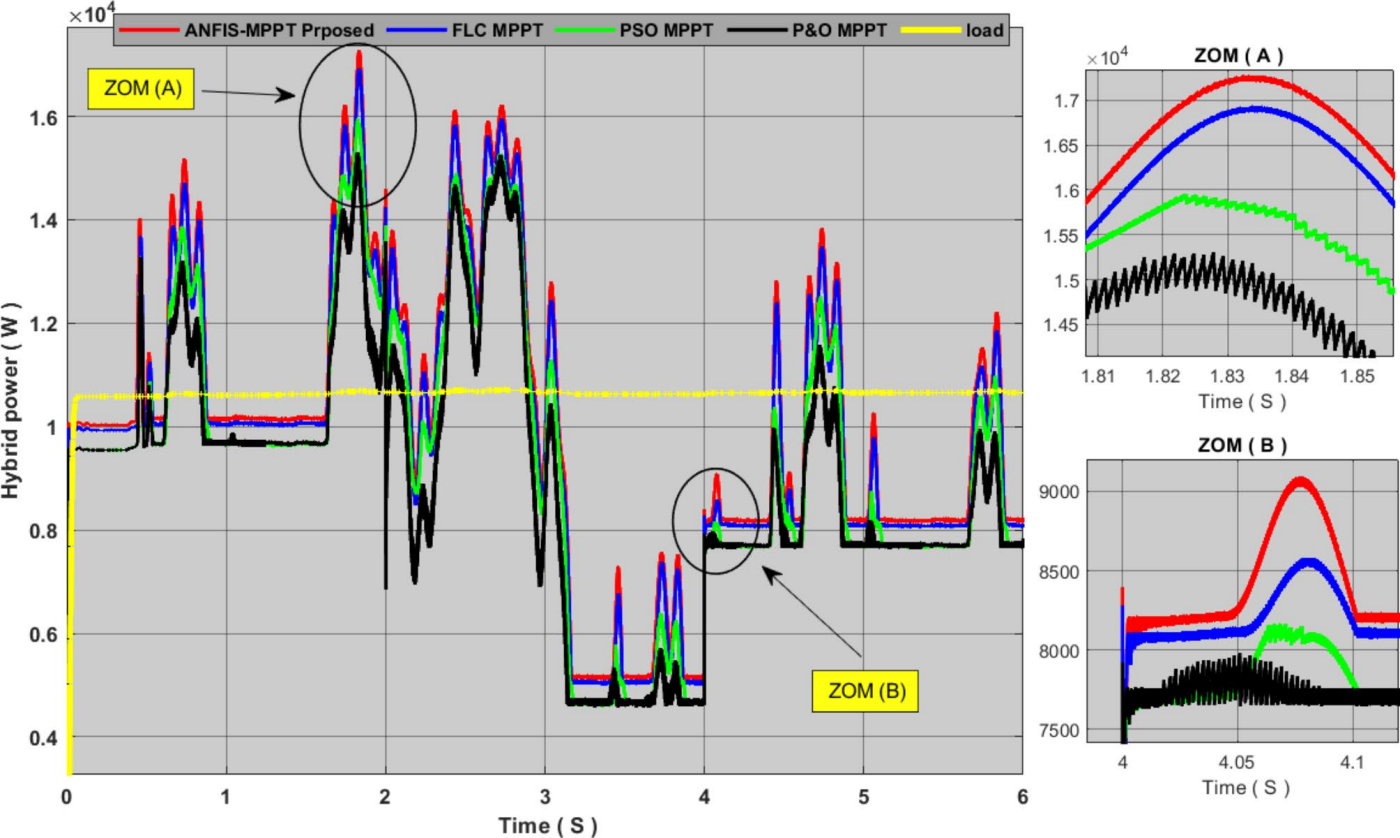
comparison between the MPPT proposed and other MPPT technique power generated by the hybrid system.

**Fig. 13:**
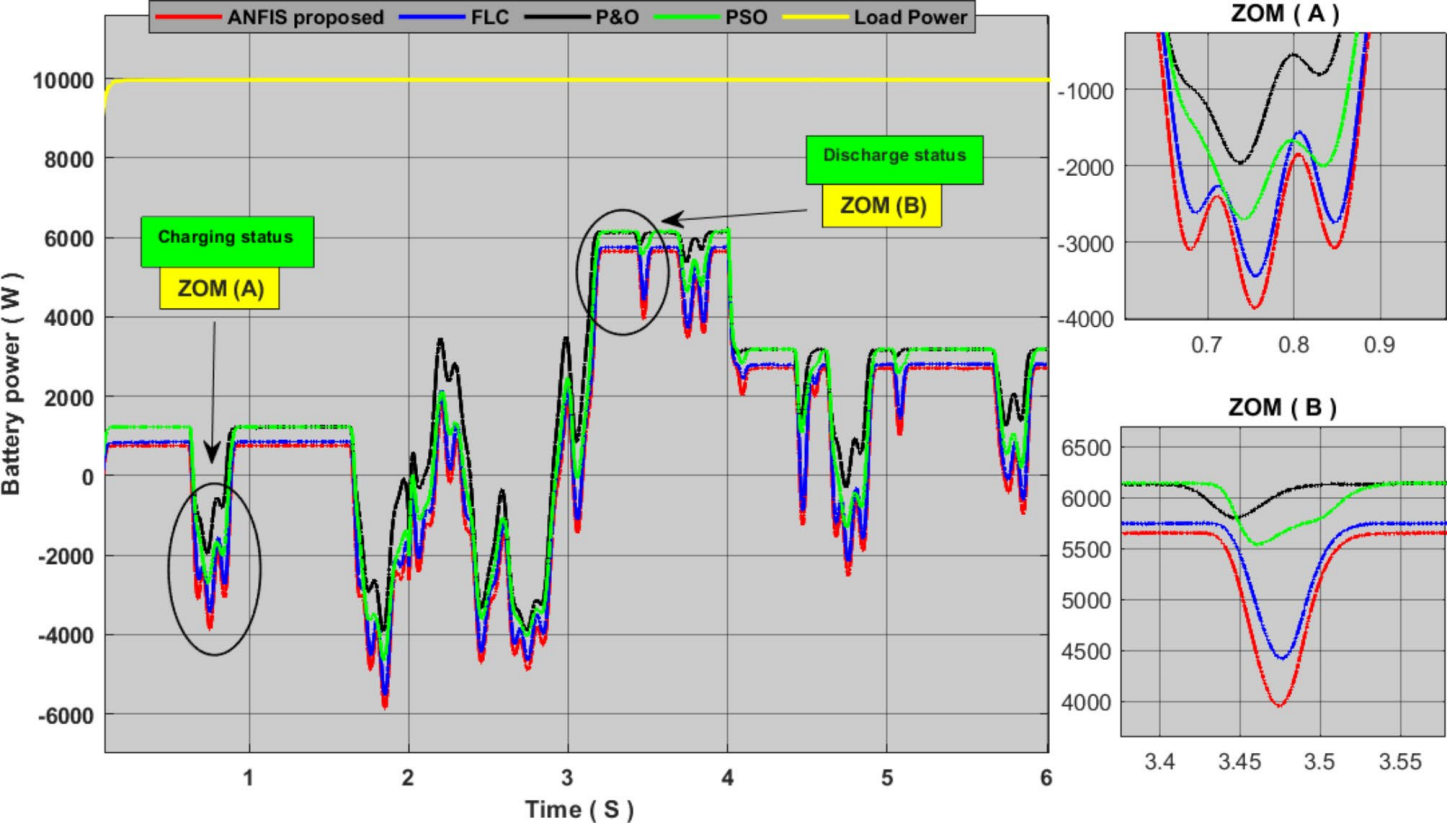
Comparison of storage system energy between the proposed MPPT and other MPPT techniques.

In order to further verify the effectiveness and performance of the proposed techniques for PVS and WECS, we combined them to form a hybrid power generation system, and we added a storage system for proper management of power flow. Figures 12 and 13 show the results of simulating this system. We can see from these results that the P&O algorithm was characterized by weak output power with slow response time and clear oscillations throughout the experiment time, and all these dynamics translate into large energy losses. The PSO technique is characterized by fewer oscillations and a greater output yield than the P&O algorithm, but it has a slow response time. This technology allows a little more energy storage than its predecessor. The FLC method has almost zero oscillations with good output yields, but as we mentioned earlier, the weakness of this technology is its slow response time. As for the proposed approach, it was found that it gave an excellent production yield with fast response time and zero oscillation, and it also contributed to storing the largest amount of energy compared to other technologies. Therefore, we can say that this approach increases the efficiency and performance of the hybrid system and also contributes to reducing the cost of production.

In hybrid systems powered by renewable energy sources, the storage system is crucial to preserving consistent and dependable power quality. Its erratic and unpredictable character is the reason behind this. To effectively regulate the bidirectional converter, this work provides an intelligent controller-based ANFIS. The suggested controller seeks to lessen DC bus oscillations and preserve voltage stability at the designated reference value of 540 V.

**Fig. 14:**
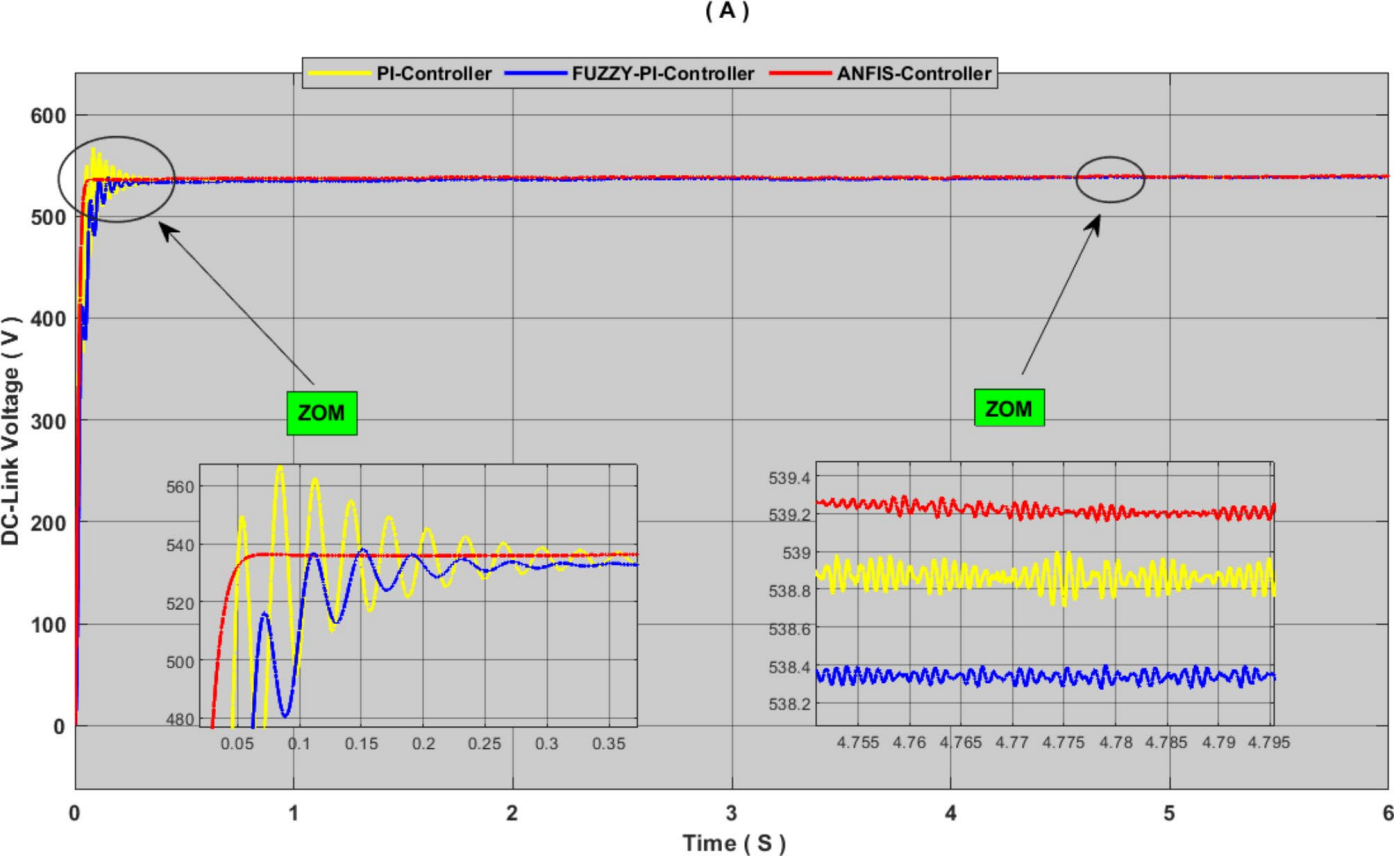
DC-link voltage, comparison between proposed controller and PI-controller and FUZZY-PI controller.

**Fig. 15:**
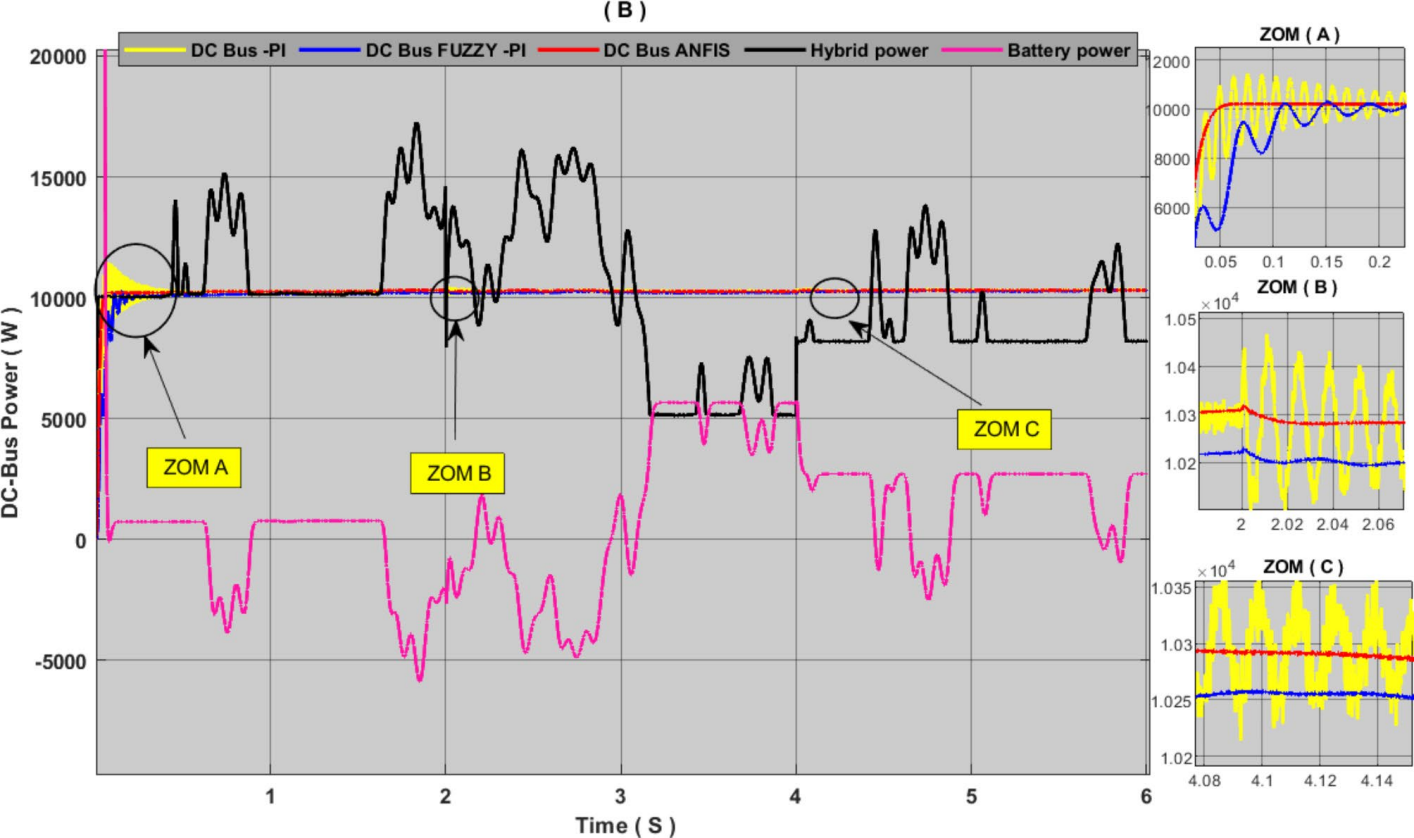
DC-link power, comparison between proposed controller and PI-controller and FUZZY-PI controller.

Figures 14 and 15 show the simulation results of DC-link voltage and DC-link power under variable power output conditions. The results were analysed through a critical comparison of the proposed ANFIS controller with PI controller and Fuzzy controller according to the value of oscillations, response time and overshot ratio, we can notice that the PI controller is characterized by clear oscillations at the DC-link and a slow response time, and the overshot ratio reached 6%. FLC is characterized by a weak overshot value with few oscillations, but the drawback that can be noticed in this controller is response time is rather slow. As for the proposed controller, it has been shown that it has a fast response time with almost no oscillation. This approach also contributed to eliminating the overshot defect, as the results.

show that its value is almost 0%. Through these results, it can be said that the proposed approach to controlling the bidirectional converter contributed to robust the performance of the hybrid system and improving the quality of the energy produced. Table 6 shows more detailed analyses and comparisons of this simulation results.

**Table 6:** Dynamics COMPARISON BETWEEN proposed Controller and other controllers.

specification	Overshoot	Oscillation	Response time
PI	6%	high	slow
Fuzzy-PI	1%	medium	medium
ANFIS-PI	0.01%	low	fast

*B. Load side power flow management*.

In this study, we conducted intelligent energy flow management based on the fuzzy logic of an environmentally friendly hybrid system. Renewable sources of power generation and battery are adopted to balance the system in cases of surplus or energy deficit. 80% percent of the battery charge value was considered the maximum value, and 20% was the minimum value. This is to conserve battery life. On the loading side, the system consists of a DC load, an AC load, and a water pump. Loads were divided into different priorities. The first basic AC load consists of 8 kW and a secondary AC load of 4 kW. Basic DC load of 4 kW, 2 kW secondary DC, and 2 kW water pump This brings the total load to 20 kW.

**Fig. 16:**
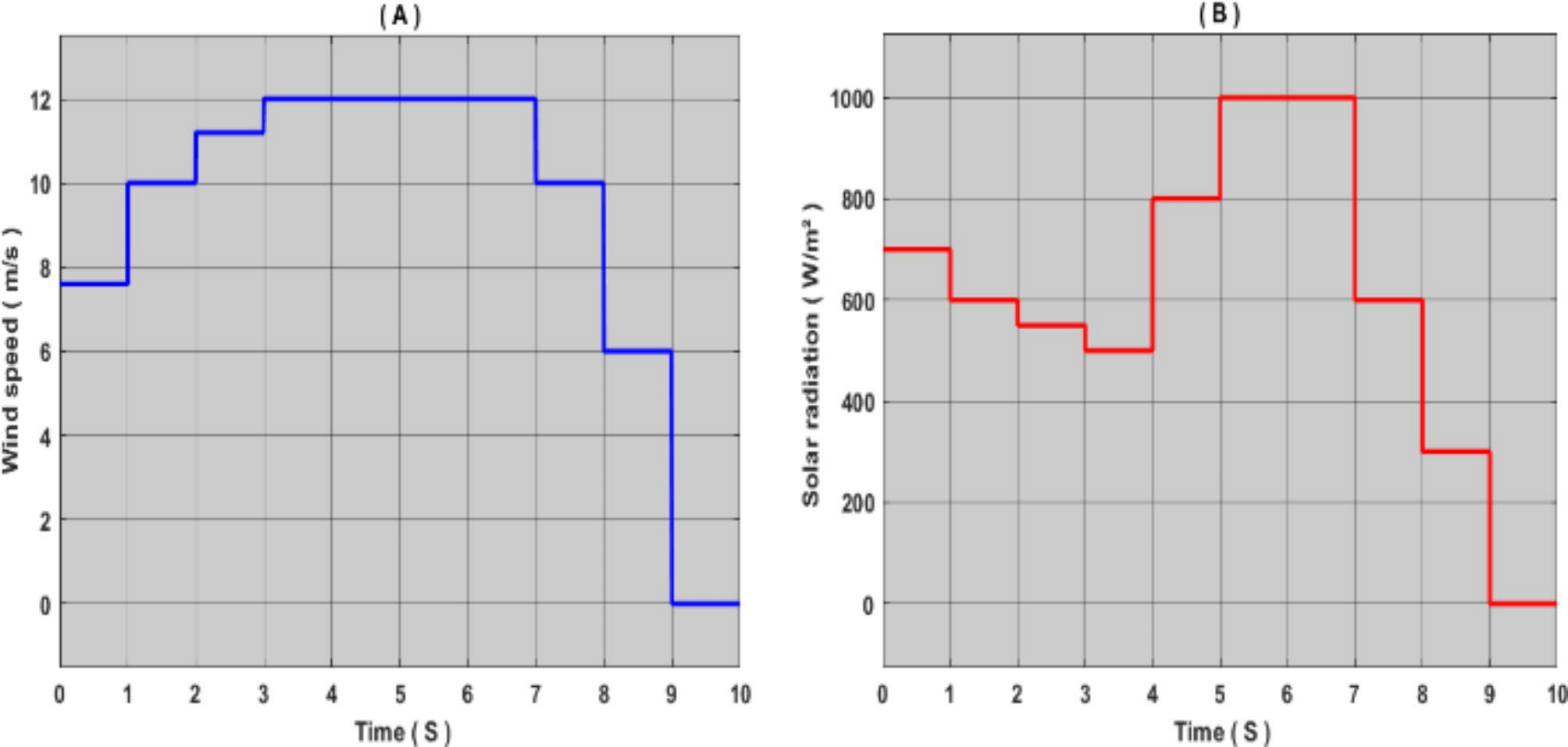
Proposed meteorological parameters: (a) wind speed, (B) sun radiation.

In order to test the proposed energy management algorithm, we gave random values for wind speed and solar radiation, Fig. 16 shows the value of solar radiation and wind speed applied to this work.

**Fig. 17:**
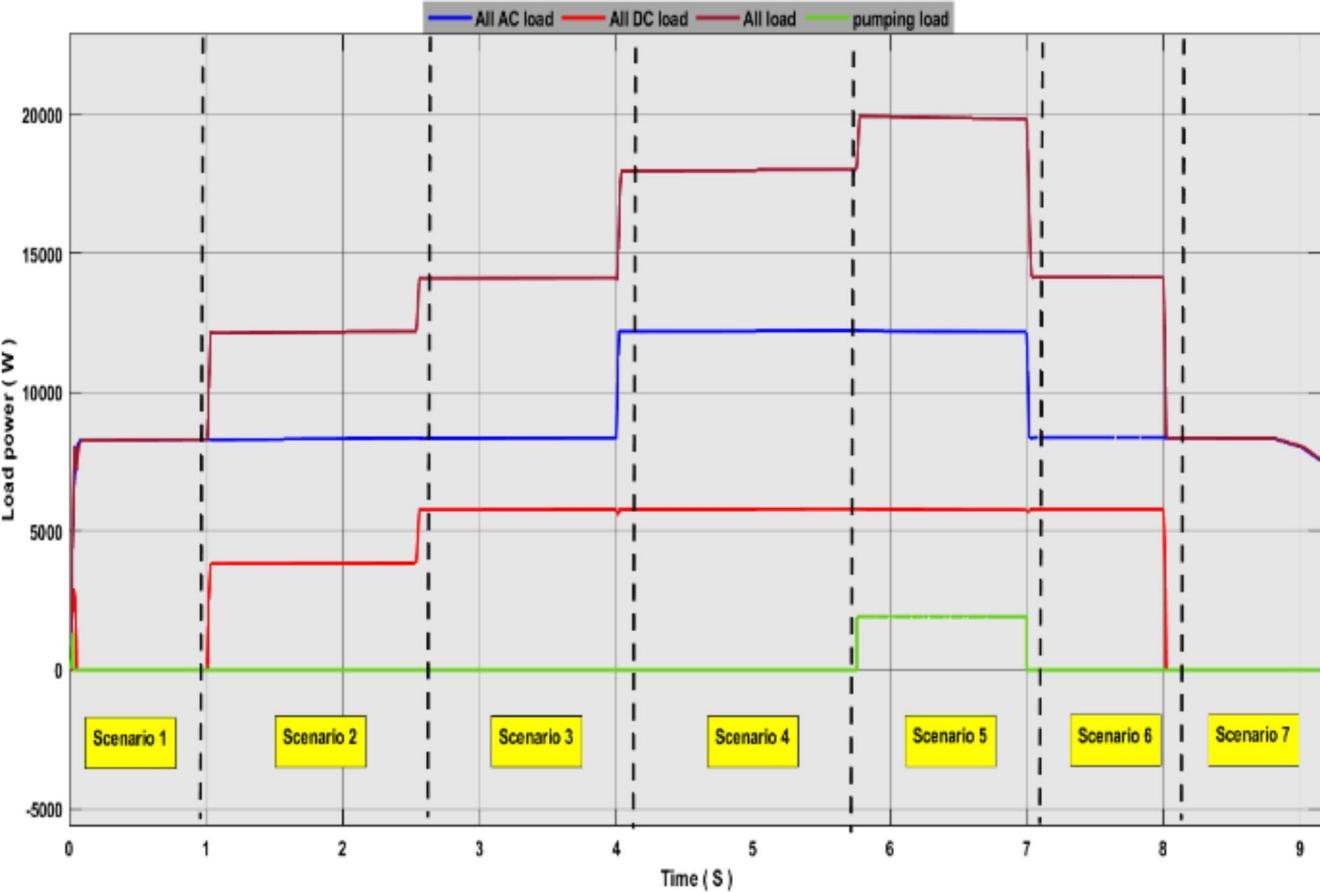
Simulation results of Load power flow management (AC load. DC load. Pumping load).

**Fig. 18:**
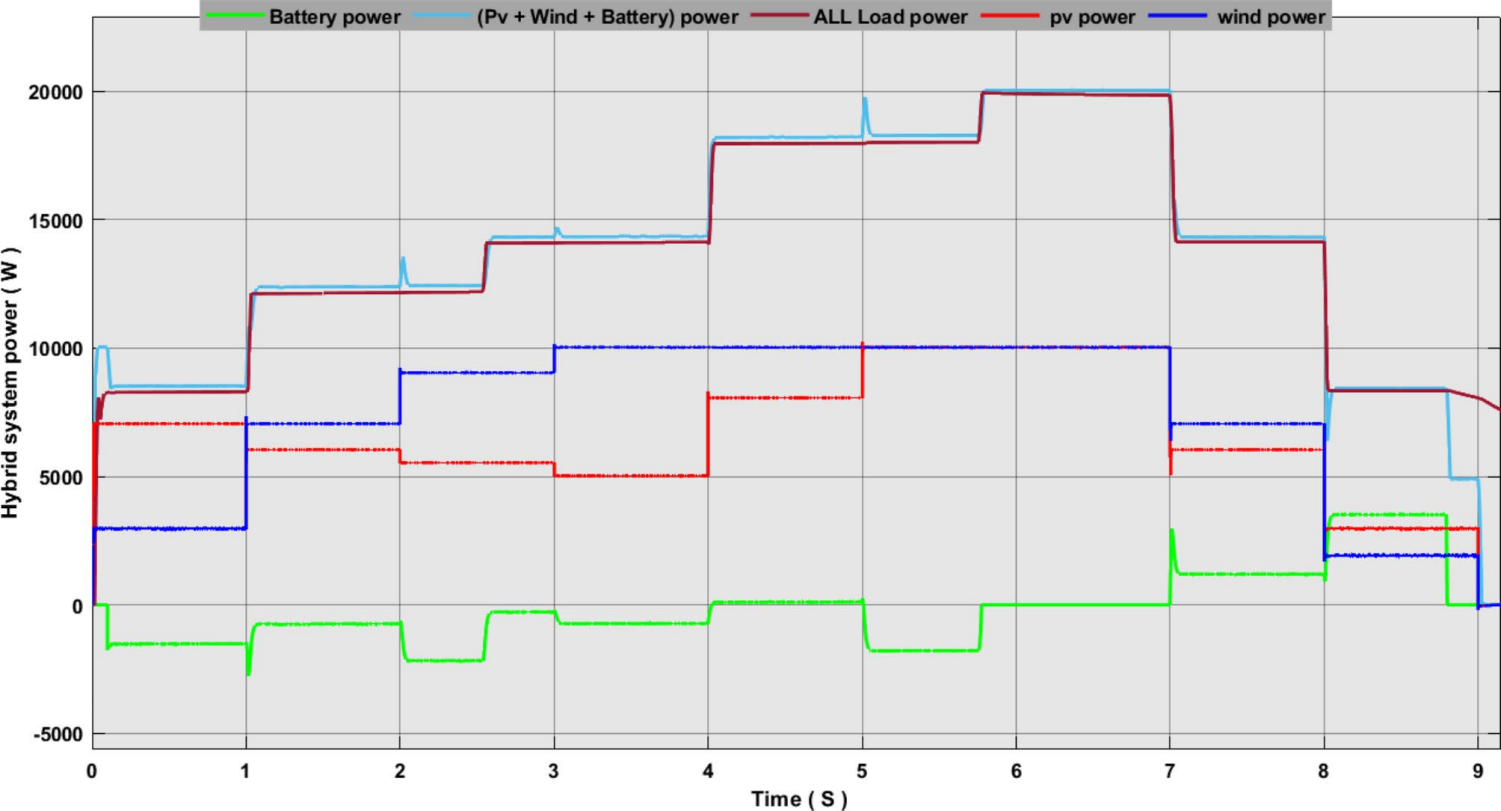
Simulation results of hybrid system power management.

**Fig. 19:**
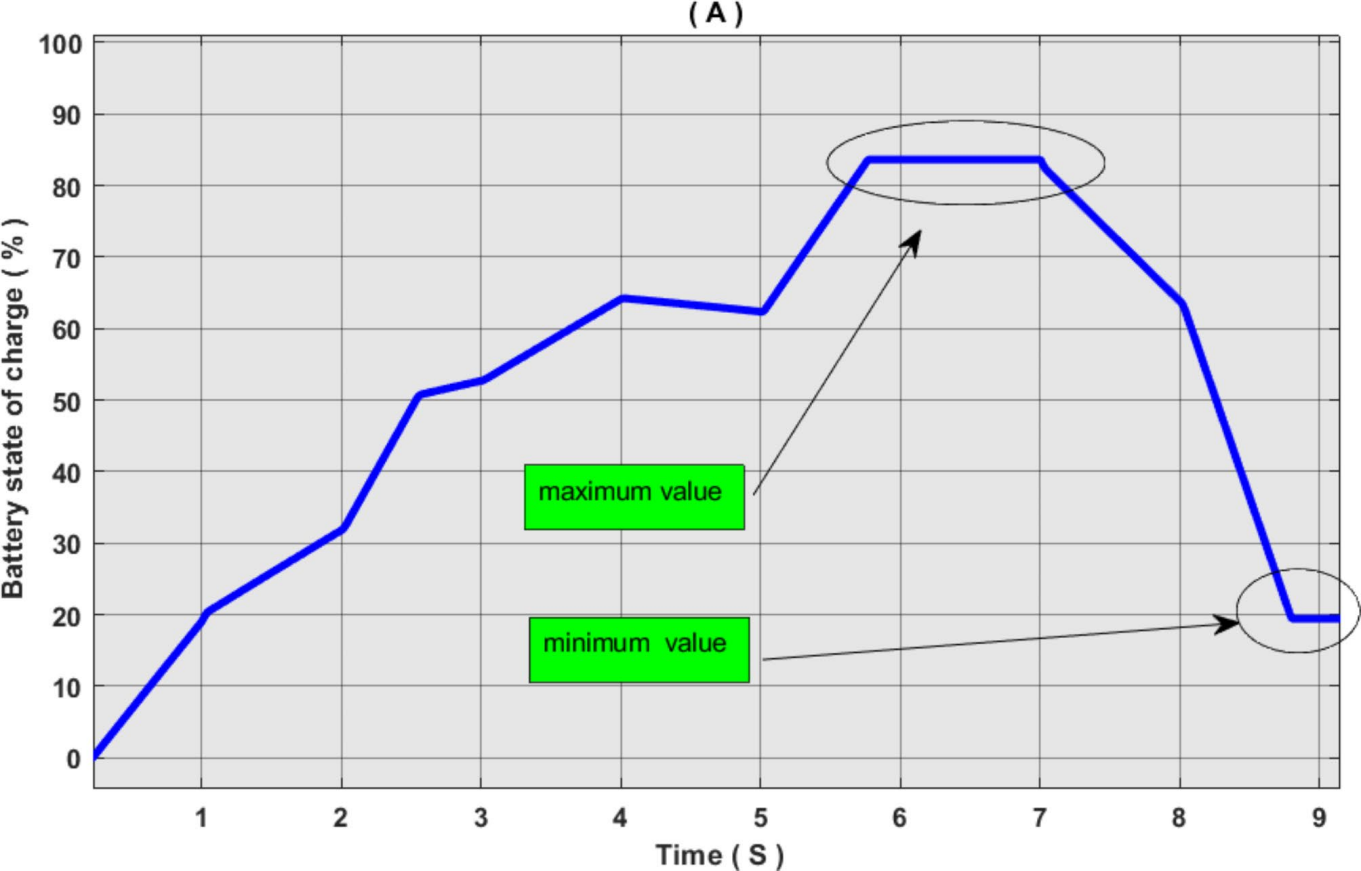
Simulation results of battery storage system, (A) battery state of charge, (B) battery current.

Figures 17, 18 and 19 show the energy flow management simulation results for the proposed system. These results show that the system has gone through many different scenarios.

(P pv, P wind, P battery, P load total) For many expected scenarios.

In the scenario 1, we observe that the controller has linked only the base load AC1, commands were given from the controller to connect this load based on the input factors. Where we see from Fig. 18 that the total energy produced from renewable sources is 10 kW while the battery charge ratio was at 0 the power was provided to the base load and 2 kW of surplus energy was charged in the battery.

In Scenario 2, we notice that the power produced increased to 13 kW and the battery charge rate exceeded 20%. This led the control unit to give orders to connect the primary DC-load, DC1.

In Scenario 3, we notice that the power produced increased to 14.5 kW, and the battery charge rate exceeded 50%. In this scenario, both DC1 and DC2 were connected, and the surplus energy was stored.

In Scenario 4, we notice that the power produced value reached 18 kilowatts and the battery charge rate exceeded 60%. In this scenario, both AC1 AC2 and DC1 DC2 were connected.

In scenario 5, we notice that the power produced value reached its peak of 20 kW, and the battery charge rate reached a maximum value of 85%. In this scenario, all loads were connected, and the battery was disconnected from the system.

In scenario 6, we notice that the power produced decreased to 13 kW and the battery charge rate decreased to 60%. In this scenario, both the pumping load and AC2 were disconnected, and the battery was connected. We notice that the battery is discharging, and this is due to its high charge percentage.

In scenario 7, the power produced decreased to 5 kW, while the battery charge percentage was greater than 20%. In this scenario, all loads were disconnected except for the AC primary load, which was powered by the battery. The battery supported the system until the charge rate reached less than 20%, at which point the battery was disconnected from the system.

## Discussion

Extensive research has been conducted on the topic of energy management for hybrid systems that rely on renewable energy sources. A wide variety of second-hand sources have been discovered in power generation and storage systems. The discussions about prior pertinent studies are succinctly described in Table 7. As for our study, we designed an independent hybrid system based on renewable sources. It consists of a photovoltaic system, wind power, and a storage system. In terms of controlling energy management in our study, the policy of splitting loads into different priorities has been used. Given the unpredictable nature of self-based renewable sources, this is the optimal approach. We have added a water pump to this work to take advantage of surplus power in extreme production cases, and in condition of a fully charged battery. The water can be utilized by storing it in tanks or using it for agricultural purposes. This is better than using discharge charges or stopping one of the generating sources from producing. In the proposed system, the battery was protected in all working conditions. It is separated if the charge ratio is 85%. and there is a surplus in power production. It is separated if the charge ratio is less than 20%. and there is a deficit in power production.

**Table 7:** A comparative study with related work.

References	Energy Source	Type of system studied	Optimization Objectives
(17)	Wind. PV Fuel cell	Micro-grid	Maintaining energy balance
(21)	Wind. PV Biomass	Grid-connected	Reducing the expenses associated with operations. Saving energy for loads
(13)	Diesel. g Wind. PV	Stand-alone	Improving effectiveness and performance
Our Work	PV Wind	Stand-alone	Improving hybrid system Efficiency Protect battery Life. Maximize the utilization of the generated energy. Minimize the release of pollutants into the environment

## Conclusion

In conclusion, the global increase in energy consumption and the great interest in renewable sources has led to a trend towards developing and improving energy production based on renewable resources.

This paper has dived into the development and improvement of the efficiency of the stand-alone hybrid system and lays a solid foundation for future improvements. The maximum possible power of the photovoltaic and wind systems can be achieved thanks to the proposed MPPT technique, which has shown good results compared with the techniques mentioned in the literature. The oscillation around the MPP were reduced and the production efficiency was improved, as the proposed technique gave a percentage of 99.7% for PVS and 98% for WECS. The response time was also improved, reaching 0.04s. One of the most important aspects of this work was confirmed: maintaining stable voltage at the DC bus, thanks to the proposed Pi-ANFIS. We verified the performance of the proposed controller under variable conditions of solar radiation, wind speed, and load changes using MATLAB/Simulink.

The second goal of this work is the intelligent management of energy flow between loads. We proposed an algorithm based on fuzzy logic to manage loads. This algorithm relies on dividing loads into priorities. This strategy showed clear superiority over traditional strategies for energy management, as the simulation results showed that it is possible to benefit from the total amount of energy produced in all weather conditions, and it can also provide energy for the primary load in all scenarios.

In future versions, this work can be developed. This system may be exploited to charge electric car stations or used to pump water to agricultural areas. It is possible to further diversify storage types and energy generation sources other than the sun and wind. MPPT technologies can also be developed and their performance improved further. Our efforts contribute to a clean and environmentally friendly future by developing energy generation systems based on environmentally friendly sources.

## Data Availability

The datasets used and/or analysed during the current study available from the corresponding author on reasonable request.
